# Anatomical Variations and Morphometry of Carotid Sinus: A Computed Tomography Study

**DOI:** 10.3390/tomography11040045

**Published:** 2025-04-07

**Authors:** Noor Fazaldad, Srinivasa Rao Sirasanagandla, Anwar Al-Shuaili, Sreenivasulu Reddy Mogali, Ramya Chandrasekaran, Humoud Al Dhuhli, Eiman Al-Ajmi

**Affiliations:** 1 Radiology Residency Program, Oman Medical Specialty Board, Al-Khoudh, Muscat 132, Oman; r20128@resident.omsb.org (N.F.); r21159@resident.omsb.org (A.A.-S.); 2 Department of Human and Clinical Anatomy, College of Medicine and Health Sciences, Sultan Qaboos University, Al-Khoudh, Muscat 123, Oman; srinivasa@squ.edu.om; 3 Lee Kong Chian School of Medicine, Nanyang Technological University, Singapore 639798, Singapore; sreenivasulu.reddy@ntu.edu.sg (S.R.M.); ramya.c@ntu.edu.sg (R.C.); 4 Department of Radiology and Molecular Imaging, Sultan Qaboos University Hospital, University Medical City, Al-Khoudh, Muscat 123, Oman; alzuhli@squ.edu.om

**Keywords:** carotid artery, atherosclerosis, morphometry, carotid sinus, variations

## Abstract

Background: The radiological evaluation of the carotid sinus (CS) anatomy and its morphometry is essentially important for various surgical procedures involving the carotid bifurcation and the CS itself. Despite its tremendous clinical significance, studies dealing with the CS anatomy are seldom reported. Hence, the present study aimed to evaluate the frequencies of the CS positional variants and their morphometry and correlate them with age and body mass index (BMI). Methods: In this retrospective cross-sectional study, a total of 754 disease-free carotid arteries were examined using computed tomography angiography scans to determine the CS positional variations (such as types I to III) and its morphometry, including the CS diameter and length. Additionally, the association between these parameters and factors such as sex, age, and body mass index were explored using appropriate statistical tests. The inter-rater agreement of the collected dataset was evaluated using Cohen’s Kappa. Results: The CS type I was observed in 87.67% of the cases, and type II and type III were observed at lower frequencies with 9.02% and 3.32%, respectively. There were statistically significant (*p* < 0.001) differences observed in the mean diameter and length of the sinus between the sex and the type I CS variations. However, there was no significant and strong correlation between the age and BMI factors with sinus length and sinus diameter. The kappa values for inter-rater agreement ranged from 0.77 to 0.99 for all parameters. Conclusions: In type I, the CS length and carotid vessel’s diameter is significantly different between the sexes. However, age and BMI do not affect the CS anatomy in radiologically disease-free carotid arteries. Knowledge of the CS variant anatomy is clinically significant as it influences the patients’ surgical and physiological outcomes.

## 1. Introduction

The carotid sinus (CS) is a visible localized normal dilatation or bulging commonly located at the arterial wall of the proximal internal carotid artery (ICA), which may extend into the common carotid artery (CCA) and/or the external carotid artery (ECA). Structurally, it has abundant elastic fibers and contains stretch receptors or baroreceptors sensitive to blood pressure (BP) variations [1,2]. The CS receptors are typically involved in the ‘baroreflex’, in which any alteration in the BP reflexly produces bradycardia and vasomotor dilatation to lower the BP. The CS neural elements are supplied by the vagus, glossopharyngeal, and sympathetic nerves [3]. These fibers carry sensory information from the CS baroreceptors to the nucleus tractus solitarius in the medulla oblongata. At the same time, the autonomic nervous system carries efferent information to reduce the heart rate and vasomotor tone [1,2]. Any structural and functional changes in the heart and kidneys result in alterations in the BP. In such cases, the carotid baroreflex comes into action to regulate the BP within normal physiological limits [2]. Clinically relevant CS variations and their associated essential nerves may be linked to altered functions such as BP and heart rate via the baroreceptors. Recognizing the aberrant CS anatomy may be critical to diagnosing and treating conditions such as CS hypersensitivity, and ensuring safe neck surgical procedures [4].

The CS location is found to be variable between individuals and within the same person [1]. Very few studies have studied the CS location variations in the existing literature [1,5]. The CS location may be observed at four different sites of the carotid arterial system [1]. The anatomical variations of the carotid anatomy are of physiological significance as they increase the risk of hemodynamic disturbances in the bifurcation region [5,6]. Hence, the morphology of the CS plays a significant role in the risk of developing atherosclerosis plaques and stroke [6]. A larger size of the CS could serve as a risk factor for atherosclerosis by increasing the oscillatory shear index [7]. A conical-shaped ICA reduces the risk of atherosclerotic restenosis [8]. A large carotid sinus, on the other hand, increases the risk of atherosclerosis plaque formation more than a small, flared sinus [9]. A recent computational simulation study explored the role of sinus length to predict the atherosclerotic plaque formation based on risk analysis parameters, such as oscillatory shear index (OSI), relative residence time (RRT), and time-averaged wall shear stress (TAWSS) [10]. The relative sinus length, expressed as *α*, is calculated as the ratio of the carotid sinus’s length to the radius of the CCA. Lower TAWSS and higher OSI and RRT values are seen at the outer wall of the artery when α is equal to 7.5, as compared to when α is equal to 3.5 [10]. In other words, the study findings revealed that individuals with a longer sinus are more prone to developing atherosclerosis, providing a possible link between the sinus length and atherosclerosis risk. However, it should be noted that the study did not provide odds ratios/relative risks (OR/RR) for the atherosclerosis and stroke risk based on patient level outcome, as the research was mainly grounded in a computational fluid dynamic simulation study design [10].

The anatomical variations of the CS are also of tremendous importance during surgical interventions, including carotid endarterectomy, carotid angioplasty, and stenting [11,12]. Incidental stretching of the CS during surgical treatment may result in marked fluctuations in post-operative blood pressure, leading to hypertension and, rarely, cardiac arrest [13,14]. Further, iatrogenic injuries in this region, in turn, pose serious post-operative complications, including hemorrhage, neck hematoma, and cerebral hyperperfusion syndrome [14]. CS stimulation was reported to cause coronary spasm and resultant myocardial infarction [15]. Understanding the CS variations is important for carotid endarterectomy, wherein the surgeon desensitizes the carotid sinus to prevent the baroreceptor stimulation [4]. Additionally, the morphometry of the CS, CCA bifurcation, ICA, ECA, and degree of tortuosity are also important for clinical interventions [5].

Despite their immense clinical significance, to date, very few studies have been reported on the CS positional variations and the morphometry of carotid arteries at the level of the CCA bifurcation [1,5]. Furthermore, the sample size used in these previous studies to determine the prevalence of different CS variants and morphometry is too small, and most of them are cadaver-based studies [1,5]. Hence, the present study aimed to evaluate the frequencies of the CS positional variants and its morphometry and correlate them with age and BMI in Omani subjects who had been referred for computed tomography angiography (CTA). CTA is considered a robust modality for assessing the parameters we aimed to evaluate in the present research.

## 2. Methodology

Study setting and population: The present study was a retrospective, cross-sectional study conducted in the Department of Radiology and Molecular Imaging at Sultan Qaboos University Hospital, a tertiary care hospital in Oman. Ethical approval was obtained from the Medical and Research Ethics Committee at the College of Medicine and Health Sciences at Sultan Qaboos University (Ref. No. SQU-EC/019/2023). This study conforms with the Declaration of Helsinki, and since it was conducted retrospectively, informed consent was waived. We screened CTA scans of adult Omani subjects (aged ≥ 18 years). The main clinical indications of the CTA examinations included in this study were stroke, transient ischemic attack, and trauma. The body mass index (BMI) was collected for the included patients.

Inclusion criteria: Omani patients of either sex, aged ≥ 18 years, who had been referred for CTA examination and had radiologically disease-free carotid arteries during the period from January 2018 to December 2022 were included.

Exclusion criteria: Patients with evidence of carotid vascular pathologies, such as atherosclerosis, tumors, inflammation, or previous surgery or intervention, were excluded. Patients under the age of 18, non-Omani patients, and examinations with significant motion artifacts were excluded from this study.

### 2.1. Image Acquisition and Data Collection for Carotid Sinus Variations and Morphometry

Every patient had a neck CTA scan utilizing a 64-slice multidetector CT scanner (Siemens Sensation 64) with a detector configuration of 64 × 0.6 mm, a pitch of 0.7, and a peak voltage of 100 kV. The scan range was from the carina to the vertex. The FUJIFILM Worldwide Picture Archiving and Communication System (PACS) (Synapse PACS, version 5.7.102) was utilized to examine the scans. The images were evaluated in the axial plane with coronal and sagittal reformats using a slice thickness of 0.75 mm. The PACS tools were employed to measure the dimensions.

**Figure 1:**
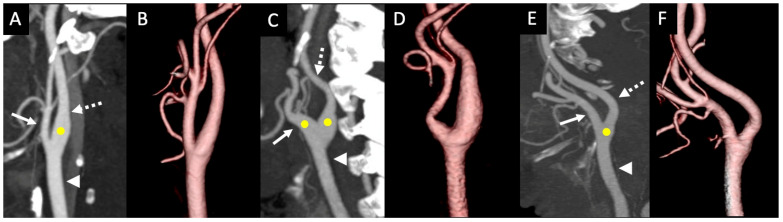
Types of carotid sinus using CT maximum intensity projection images and CT 3D reconstructions: (**A**,**B**) type I with dilatation of the CS extending from the distal CCA to the ICA; (**C**,**D**) type II with a dilated CCA extending to both the ICA and ECA; (**E**,**F**) type III CS with the dilatation in the distal CCA without extension to the ICA or ECA. (Arrowhead = CCA, solid arrow = ECA, dashed arrow = ICA, yellow circle = extension of the CS).

The CS positional variations were documented from type I to type IV, as described previously by West et al. (2018) (Figure 1) [1]. In type I, the CS is located in the CCA and extends into the ICA; in type II, the CS begins at the CCA and extends into both the ECA and ICA; in type III, the CS is located only at the CCA without any extension into either of its terminal branches; in type IV, the CS extends from the CCA to the initial segment of the ECA (Figure 1). Using the features in PACS, two radiology residents measured independently the maximum diameter of the terminal portion of the CCA and the initial segments of its terminal branches; the ICA and ECA were measured at the carotid artery bifurcation. Most importantly, the vessel with the maximum diameter of the CS was measured. The maximum diameter of the vessels was measured in the axial plane after correction for any obliquity. The length of the CS was recorded as described previously by Baz et al. (2022) [5]. The CS sinus length in the type III variant was not measured because there is a chance of minimal sinus extension into the terminal branches.

### 2.2. Statistical Analysis

The data analysis was performed using SPSS, version 29 (IBM Corp., Armonk, NY, USA). The descriptive statistics were used to present the CS positional variations, sinus length, and diameter. The Kolmogorov–Smirnov test was performed to assess the normality of the dataset. A chi-squared test was used to compare the CS positional differences with sex and laterality. An independent *t*-test was used to compare the means of the carotid vessels’ diameters, the CS diameter, and the sinus length based on sex and laterality. This means multiple *t*-tests were conducted, comparing the sex and laterality across the carotid vessel’s diameters (12 comparisons), CS length (3 comparisons), CS diameter (2 comparisons) among three different carotid sinus types, necessitating Bonferroni adjustment to mitigate the type I error rate. Thus, the adjusted alpha level for the carotid vessel’s diameters, the CS length, and the diameter were 0.004, 0.017, and 0.025, respectively. The effect size was calculated using Cohen’s d for the statistically significant comparisons. The data was presented as a mean (median) ± standard deviation. To determine the changes in the carotid vessels and the carotid sinus related to age, the cases were divided into three groups: 18–39, 40–69, and ≥70. A one-way ANOVA was used to compare these groups. Pearson’s correlation test was employed to verify the extent of the association between age and BMI concerning vessel diameter, as well as the CS length and diameter. The inter-rater agreement of the collected dataset was evaluated using Cohen’s kappa [16]. The kappa values were interpreted as follows: ≤0 = no agreement; 0.01–0.20 = no agreement to slight agreement; 0.21–0.40 = fair agreement; 0.41–0.60 = moderate agreement; 0.61–0.80 = substantial agreement; and 0.81–1.00 = almost perfect agreement. A *p* value of less than 0.05 was considered statistically significant.

## 3. Results

Demographics: A total of 754 carotid arteries were examined for the morphometry of the CS and its positional variations. The average age of the patients included in this study was 49.00 years ± 15.35. This study included 56.7% (428/754) of men’s carotid arteries and 43.2% (326/754) of women’s carotid arteries. There were 50.39% (380/754) right-sided CTAs and 49.60% (374/754) left-sided CTAs. The K-S test was performed, and the distribution was normal in both sex and age categories. Therefore, the mean differences were evaluated using the independent sample *t*-test or ANOVA, as appropriate.

The CS positional variations: The classical CS positional type I was observed in 87.67% (661/754) of the cases. The rest of the types were observed at lower frequencies, with 9.02% (68/754) being type II and 3.32% (25/754) being type III. The studied sample did not identify a type IV of the CS. Table 1 presents the overall frequency of the different types of CS, as well as sex and laterality. In men and women, the classical type (type 1) was observed in 89.72% and 84.97% of the cases, respectively, which was found to be statistically significant (*p* < 0.001). Similarly, the classical type I CS was observed in 89.47% on the right side and 85.83% on the left side, which seems insignificant (Table 1). No statistically significant differences were observed when other CS positional variations were compared for sex and laterality (*p >* 0.05).

The diameter of the carotid arteries and the CS length based on sex: The mean diameter of the ICA, ECA, and CCA at the level of CCA bifurcation in the different types of CS in men and women is presented in Table 2. The overall diameter of the ICA for type I in men was 7.53 ± 0.04 mm, and was 6.76 ± 0.06 for women. The diameter of the ECA in type I for men and women was 5.05 ± 0.37 mm and 4.47 ± 0.04 mm, respectively. On the other hand, the diameter of the CCA in type I for men and women was 9.54 ± 0.64 mm and 8.60 ± 0.07 mm, respectively. Significant sex differences were observed in the diameters of the ICA (*p* < 0.001), the ECA (*p* < 0.001), and the CCA (*p* < 0.001) in the type I CS with large effect size (Table 2). The sex differences in mean sinus length and the mean diameter of the sinus are presented in Table 2.

In men, the mean length of the CS in type I was 12.72 ± 0.08 mm, and in type II was 13.72 ± 0.311. In women, the mean length of the CS in type I was 11.75 ± 0.09 mm, and in type II was 12.92 ± 0.23 mm. Significant sex differences were observed in sinus length for the type I CS (*p* < 0.001) with large effect size. While considering all types, the vessel with a maximum diameter at the sinus was found to be the CCA with 99.1% (747/754) frequency, followed by the ICA with 0.9% (7/754) frequency. The mean diameter of the CS located in the CCA was significantly higher in men than in women (*p* < 0.001; Table 2) with large effect size.

The diameter of the carotid arteries and the CS length based on laterality: Table 3 presents the laterality differences in the mean maximum diameter of the ICA, ECA, and CCA, and the mean CS length and diameter. There were no statistically significant differences observed between the sides in all these parameters (Table 3; *p* > 0.05).

The diameter of the carotid arteries and the CS length based on age groups: The parameters, including the mean maximum diameter of the ICA, ECA, and CCA at the level of the CCA bifurcation, mean sinus length, and mean diameter of the CS, were compared across various age groups; the data are presented in Table 4. No significant differences were observed in these parameters between different age groups (one-way ANOVA; Table 4).

A Pearson correlation analysis was used to measure the strength of the relationship between age and BMI versus their sinus length and in type I. A weak negative correlation with no statistically significant differences was observed in both age versus sinus length (r = −0.067; n = 321) and BMI versus sinus length (r = −0.026; n = 384). However, a weak positive correlation was observed in both age versus mean sinus diameter (r = 0.067; n = 210) and BMI versus mean sinus diameter (r = 0.172; *p* < 0.01; n = 438). The kappa values of inter-rater agreement for all the parameters, including positional variations, carotid vessel diameters, sinus length, and sinus diameters, ranged between 0.77 to 0.99, indicating a good agreement between the observers.

## 4. Discussion

The carotid arterial system shows variations in common carotid artery bifurcation angles, level of bifurcation, and the CS location [1,17]. These variations may occur between the individuals and within the same individual. Sex differences in the frequencies were also reported [5,17]. The carotid arterial system variations could be attributed to errors in the normal development of these vessels. The ICA and CS are developed from the third aortic arch, particularly the distal segment. The CS appears from the ectodermal sheet during childhood. However, starting only from the teenage years is the complete CS present at the base of the ICA [18,19]. The strength of this study lies in its large population size and the involvement of two independent raters, which provides more reliable and valid results regarding the CS variation frequencies and morphometry. The data obtained from this study, especially the morphometry and positional variations of the CS, are clinically significant for performing minimally invasive treatments and surgical interventions, such as vascular clamp application, carotid endarterectomy, and carotid stenting.

Historically, there has been a discrepancy in frequencies of the CS locations in cadaveric studies. A cadaveric study by West et al. (2018) [1], using a relatively small sample, identified the CS positional variations and categorized them into four different types [1]. Among these four types, type I is considered the classical type, and the other types occur rarely and are considered anatomical variants. In this cadaveric study, CS type I, type II, type III, and type IV were observed with frequencies of 74.3%, 7.32%, 17.1%, and 1.22%, respectively [1]. In a recent imaging study, the classical type I was observed in 80% of the cases, while type II was observed in 8% of the cases, and type III was observed in 12% of the cases [5]. This study did not find type IV variations in the study population [5]. Similarly, the present study identified the classical CS type I as the predominant type in 87.67% of the cases. However, this frequency is relatively higher compared to other studies. The larger sample size in this study provides a more robust frequency estimate, compared to previous studies with smaller samples. Other factors, such as ethnic differences, systematic data collection, and higher inter-rater reliability, could account for the higher frequencies observed.

The CS is the most common site for atheromatous plaque formation, as carotid bifurcation zonal anatomy favors turbulent blood flow and reduces the shear stresses [20,21]. The CS is also a frequent site for the development of unique tumors of paragangliomas originating from the carotid bodies [22]. In severe cases of carotid sinus syndrome (CSS), surgical denervation at the CS is usually preferred to relieve the symptoms. In these patients, the diagnosis of CS hypersensitivity is performed by carotid sinus massage by palpating it externally to stimulate the baroreceptors [23]. Hence, the knowledge of positional variations of the CS reported in the present study is clinically important for diagnosing and treating the above-mentioned conditions. Most existing clinical studies of atherosclerotic carotid disease mainly focus on the type I pattern of the CS [5]. Since it is evident that, in at least 13–20% of the cases, the CS may not extend to the ICA, more studies are needed to explore the atherosclerotic pattern of carotid artery disease in other CS types [5]. In patients suffering from an oversensitive CS baroreflex, adventitial stripping of the ICA is performed to cut the afferent nerve fibers and reduce the symptoms, such as dizziness and syncope. This technique may not be successful, causing recurrence, as the CS, in a few cases, extends to the CCA and ECA. In support of this, one of the histological studies found nerve fibers of baroreflex in both the CCA and ECA [3]. The positional variants and morphometry reported in the present research may offer helpful insights into the successful planning of surgeries in patients with an oversensitive CS baroreflex.

In addition to the positional variations, the morphometry of the CS, including its length and diameter, is clinically important for the successful planning of surgical procedures, including carotid endarterectomy, carotid angioplasty, and stenting, among other procedures. Imaging studies have advantages over cadaveric studies in providing accurate values. To date, only one study has explored the morphometry of the CS and correlated the dimeter of the sinus with the position and size of the sinus [5]. This study’s findings indicate that the mean diameter of the CS located on the ICA was 8.02 mm in men and 6.27 in women; while, on the CCA, it was 7.9 in men and 6.99 in women, showing significant sex differences [5]. Similarly, in the present study, the CS diameter is significantly higher in men with large effect size. In the same study, the mean diameters of the CCA, ICA, and ECA were 7.39 ± 1.04 mm, 6.71 ± 1.49 mm, and 4.27 ± 0.75 mm, respectively [5]. These values are comparable to the present study measurements. Similarly, in this study, no laterality or age differences were observed in vessel diameters. Additionally, similar to the previous study [5], the length of the CS was significantly higher in men than in women in a type I sinus with large effect size. Thus, this study’s findings highlight the importance of considering sex differences in assessing the sinus dimensions and their potential implication for carotid sinus health.

Our analysis also focused on correlating the BMI and age factors with the sinus length and diameters. Although there were no significant differences in a previous study, a positive correlation was observed between the sinus length and age [5]. By contrast, our study found a weak negative correlation between the sinus length and age and BMI. Previously, it has been demonstrated that the carotid vessels’ diameters at bifurcation increase with age. However, such an increase depends on vascular risk factors, including hypertension [24,25,26]. In the present study, a positive correlation was identified between the age or BMI and the sinus diameter. This positive correlation indicates that sinus diameter is higher in the elderly population and those with high BMI. It has been reported that the position of the CS away from the level of the carotid bifurcation and a relatively large diameter increase the risk of developing atherosclerosis by increasing the recirculation and wall shear stress values [27]. A recent study demonstrated that increased sinus length promotes the risk of developing atherosclerotic plaque [10]. The morphometry values of the CS reported in the present study could help in predicting the risk of atherosclerosis development. However, a future study exploring the odds ratio or relative risk for the stroke or atherosclerosis end point for each type of the carotid sinus is warranted.

The present study has the following limitations. This study is a retrospective study limited to the Omani adult population data set from a single tertiary care hospital in Oman. The current study involved the normal CS based on the radiological assessment and did not evaluate the CS morphology and morphometry changes over time. A future study design involving longitudinal patient-based imaging, clinical data, and outcome tracking to determine the association of carotid sinus pattern/morphology on the development of the sinus pathologies is warranted.

## 5. Conclusions

This study, for the first time, reports the positional variations and morphometry of the CS, including the length and diameter, in a relatively large sample. The type I CS was the most prevalent and classical type with 87.67% of the cases. There were significant sex differences in the sinus diameter and sinus length in the type I CS of the study subjects based on the anatomical measurements. However, the clinical and therapeutic relevance of such differences needs to be further evaluated in future studies. There was no significantly strong correlation between the age and BMI factors and the sinus length and diameter. The knowledge of anatomical variants and morphometry of the CS is clinically important for surgeons and interventional radiologists when performing various surgical procedures, such as vascular clamp application, carotid endarterectomy, and carotid stenting.

## Figures and Tables

**Table 1:** The sex and laterality differences in carotid sinus positional variations.

Variable	CS Type I	CS Type II	CS Type III
Total (n = 754)	87.67 (661/754)	9.02 (68/754)	3.32 (25/754))
Men (n = 428)	89.72 * (384/428)	7.94 (34/428)	2.34 (10/428)
Women (n = 326)	84.97 * (277/326)	10.43 (34/326)	4.60 (15/326)
Right side (n = 380)	89.47 (340/380)	6.58 (25/380)	3.95 (15/380)
Left side (n = 374)	85.83 (321/374)	11.5 (43/374)	2.67 (10/374)

* *p* < 0.001; Chi-squared test. (CS: carotid sinus).

**Table 2:** The sex differences for various parameters—the carotid vessel’s diameter (ICA, ECA, CCA), the sinus length, and the vessel with maximum diameter at the sinus.

Vessel Max Diameter	Men (n = 428)	Women (n = 326)	*p* Value *	Effect Size
ICA				
Type I	7.53 ± 0.04 (n = 384)	6.76 ± 0.06 (n = 277)	<0.001 ^#^	15.10
Type II	8.04 ± 0.23 (n = 34)	7.57 ± 0.20 (n = 34)	0.13	NA
Type III	5.92 ± 0.14 (n = 10)	6.13 ± 0.39 (n = 15)	0.67	NA
Overall	7.53 ± 0.05 (n = 428)	6.81 ± 0.06 (n = 326)	<0.001 ^#^	13.03
ECA				
Type I	5.05 ± 0.37 (n = 384)	4.47 ± 0.04 (n = 277)	<0.001 ^#^	13.04
Type II	5.89 ± 0.15 (n = 34)	5.69 ± 0.17 (n = 34)	0.38	NA
Type III	4.78 ± 0.31 (n = 10)	4.77 ± 0.21 (n = 15)	0.98	NA
Overall	5.11 ± 0.04 (n = 428)	4.62 ± 0.04 (n = 326)	<0.001 ^#^	12.25
CCA				
Type I	9.54 ± 0.64 (n = 384)	8.60 ± 0.07 (n = 277)	<0.001 ^#^	2.06
Type II	10.79 ± 0.23 (n = 34)	9.93 ± 0.20 (n = 34)	0.01	NA
Type III	8.70 ± 0.34 (n = 10)	9.02 ± 0.42 (n = 15)	0.59	NA
Overall	8.76 ± 0.07 (n = 428)	9.61 ± 0.06 (n = 326)	<0.001 ^#^	13.04
Sinus length				
Type I	12.72 ± 0.08 (n = 384)	11.75 ± 0.09 (n = 277)	<0.001 ^#^	11.39
Type II	13.72 ± 0.311 (n = 34)	12.92 ± 0.23 (n = 34)	0.04	NA
Overall	12.80 ± 0.07 (n = 418)	11.88 ± 0.086 (n = 311)	<0.001 ^#^	11.41
Vessel with max diameter at sinus				
CCA	9.64 ± 0.06 (n = 423)	8.77 ± 0.07 (n = 324)	<0.001 ^#^	13.34
ICA	8.42 ± 0.40 (n = 5)	7.30 ± 0.40 (n = 2)	0.17	NA

* *p* value calculated using independent *t*-test and ^#^ indicates the statistically significant alpha after Bonferroni’s correction. The effect size was calculated using Cohen’s d. (ICA: internal carotid artery; CCA: common carotid artery; ECA: external carotid artery).

**Table 3:** The laterality (right vs. left) differences for various parameters—the carotid vessel’s diameter (ICA, ECA, CCA), the sinus length, and the vessel with maximum diameter at the sinus.

Vessel Max Diameter	Right Side (n = 380)	Left Side (n = 374)	*p* Value ^#^
ICA			
Type I	7.15 ± 0.06 (n = 340)	7.27 ± 0.06 (n = 321)	0.18
Type II	7.84 ± 0.30 (n = 25)	7.78 ± 0.18 (n = 43)	0.85
Type III	6.05 ± 0.33 (n = 15)	6.04 ± 0.36 (n = 10)	0.99
Overall	7.15 ± 1.22 (n = 380)	7.30 ± 1.56 (n = 374)	0.10
ECA			
Type I	4.85 ± 0.42 (n = 340)	4.75 ± 0.04 (n = 321)	0.08
Type II	5.67 ± 0.20 (n = 25)	5.86 ± 0.14 (n = 43)	0.44
Type III	4.97 ± 0.25 (n = 15)	4.47 ± 0.17 (n = 10)	0.11
Overall	4.92 ± 0.81 (n = 380)	4.87 ± 0.84 (n = 374)	0.48
CCA			
Type I	9.10 ± 0.07 (n = 340)	9.19 ± 0.07 (n = 321)	0.38
Type II	10.32 ± 0.30 (n = 25)	10.38 ± 0.19 (n = 43)	0.53
Type III	9.02 ± 0.39 (n = 15)	8.70 ± 0.42 (n = 10)	0.59
Overall	9.18 ± 1.36 (n = 380)	9.32 ± 1.34 (n = 374)	0.17
Sinus length			
Type I	12.28 ± 0.09 (n = 340)	12.34 ± 0.09 (n = 321)	0.63
Type II	13.48 ± 0.41 (n = 25)	13.22 ± 0.20 (n = 43)	0.53
Overall	12.37 ± 0.88 (n = 365)	12.45 ± 0.08 (n = 364)	0.50
Vessel with max diameter at sinus			
CCA	9.20 ± 0.07 (n = 377)	9.33 ± 0.07 (n = 370)	0.16
ICA	8.07 ± 0.88 (n = 3)	8.13 ± 0.22 (n = 4)	0.94

^#^*p* value calculated using independent *t*-test. (ICA: internal carotid artery; CCA: common carotid artery; ECA: external carotid artery).

**Table 4:** Comparison of different age groups for various parameters—the carotid vessel’s diameter (ICA, ECA, CCA), the sinus length, and the vessel with maximum diameter at the sinus.

Vessel Max Diameter	Age Groups			
ICA	18–39 (n = 210)	40–69 (n = 486)	≥70 (n = 58)	*p* Value *
Type I	7.13 ± 0.08 (n = 191)	7.25 ± 0.056 (n = 422)	7.09 ± 0.15 (n = 48)	0.36
Type II	7.02 ± 0.78 (n = 10)	7.95 ± 1.33 (n = 54)	10.48 ± 1.66 (n = 4)	0.11
Type III	6.38 ± 1.77 (n = 9)	5.76 ± 0.81 (n = 10)	6.03 ± 0.66 (n = 6)	0.56
Overall	7.09 ± 1.17 (n = 210)	7.30 ± 1.20 (n = 486)	7.03 ± 1.07 (n = 58)	0.05
ECA				
Type I	4.79 ± 0.06 (n = 191)	4.82 ± 0.03 (n = 422)	4.73 ± 0.09 (n = 48)	0.75
Type II	5.85 ± 1.25 (n = 10)	5.81 ± 0.91 (n = 54)	5.32 ± 0.57 (n = 4)	0.60
Type III	5.25 ± 0.91 (n = 9)	4.22 ± 0.57 (n = 10)	4.97 ± 0.75 (n = 6)	0.02
Overall	4.86 ± 0.85 (n = 210)	4.92 ± 0.83 (n = 486)	4.80 ± 0.69 (n = 58)	0.49
CCA				
Type I	9.15 ± 0.096 (n = 191)	9.18 ± 0.063 (n = 422)	8.86 ± 0.17 (n = 48)	0.29
Type II	10.20 ± 1.26 (n = 10)	10.38 ± 1.35 (n = 54)	10.48 ± 1.66 (n = 4)	0.91
Type III	9.17 ± 1.71 (n = 9)	8.47 ± 1.44 (n = 10)	9.18 ± 0.82 (n = 6)	0.50
Overall	9.20 ± 1.36 (n = 210)	9.29 ± 1.36 (n = 486)	9.01 ± 1.35 (n = 58)	0.26
Sinus length				
Type I	12.43 ± 1.62 (n = 191)	12.30 ± 1.57 (n = 422)	11.97 ± 1.41 (n = 48)	0.20
Type II	12.61 ± 1.16 (n = 10)	13.53 ± 1.68 (n = 54)	12.30 ± 0.96 (n = 4)	0.11
Overall				
Vessel with max diameter at sinus				
CCA	9.22 ± 1.37 (n = 210)	9.31 ± 1.38 (n = 480)	9.08 ± 1.22 (n = 57)	0.43
ICA	-	8.05 ± 1.01 (n = 6)	8.40 ± 0.00 (n = 1)	0.76

* *p* value calculated using one-way ANOVA. (ICA: internal carotid artery; CCA: common carotid artery; ECA: external carotid artery).

## Data Availability

Anonymized data are available upon reasonable request to the corresponding author.
